# Prolonged Attachment of Rhipicephalus microplus Tick Without Systemic Manifestations: A Case Report

**DOI:** 10.7759/cureus.95616

**Published:** 2025-10-28

**Authors:** Meshal M Alhameedy, Hanadi Almutairi, Ahmed Alqefari, Abdullah N Alsaleam, Eman Alsayeh

**Affiliations:** 1 Dermatology, King Fahd Specialist Hospital, Buraydah, SAU; 2 Medicine and Surgery, Qassim University, Buraydah, SAU; 3 College of Medicine, King Fahad Specialist Hospital, Riyadh, SAU

**Keywords:** arthropod bites, case report, dermatology, eosinophilic dermatitis, prolonged attachment, rhipicephalus microplus, tick bite

## Abstract

*Rhipicephalus microplus*, commonly known as the Asian blue tick, is a cattle parasite rarely reported to infest humans. We describe a 70-year-old Saudi man who presented with a month-long, asymptomatic tick attachment on his left flank after a camping trip. Examination revealed a live, engorged tick with mild surrounding erythema and edema but no systemic symptoms. Laboratory investigations were unremarkable, and histopathology of a punch biopsy showed eosinophilic dermatitis consistent with a localized hypersensitivity reaction. The tick was removed intact and identified as *R. microplus* by the regional public health laboratory. Although this species is not recognized as a human pathogen, a 14-day course of doxycycline was prescribed as a precaution. At two-week follow-up, the lesion had completely healed, and the patient remained free of systemic illness.

This case illustrates that even non-pathogenic tick species can remain attached to human hosts for prolonged periods and provoke localized inflammatory responses, underscoring the importance of careful tick removal, species identification, and individualized management of prolonged tick bites.

## Introduction

Ticks are obligate hematophagous ectoparasites that infest a wide range of vertebrate hosts, including humans. They are vectors of various pathogens, leading to significant health concerns globally [1]. Among these, *Rhipicephalus microplus*, commonly known as the cattle tick or Asian blue tick, is predominantly recognized for its impact on livestock, especially cattle, due to its role in transmitting diseases such as bovine babesiosis and anaplasmosis [2]. While *R. microplus* primarily parasitizes cattle, instances of human infestation, though rare, have been documented [3]. Such occurrences are uncommon and often underreported, leading to a limited understanding of the clinical implications of human infestations by this species. The clinical manifestations in humans can vary, ranging from mild local reactions to more severe systemic responses, depending on factors such as the duration of attachment and individual host susceptibility [4,5]. This case report presents an unusual instance of prolonged attachment of *R. microplus* to a human host without the development of systemic symptoms. The case underscores the need for increased awareness and documentation of such occurrences to enhance understanding and management of tick infestations in humans.

## Case presentation

**Figure 1:**
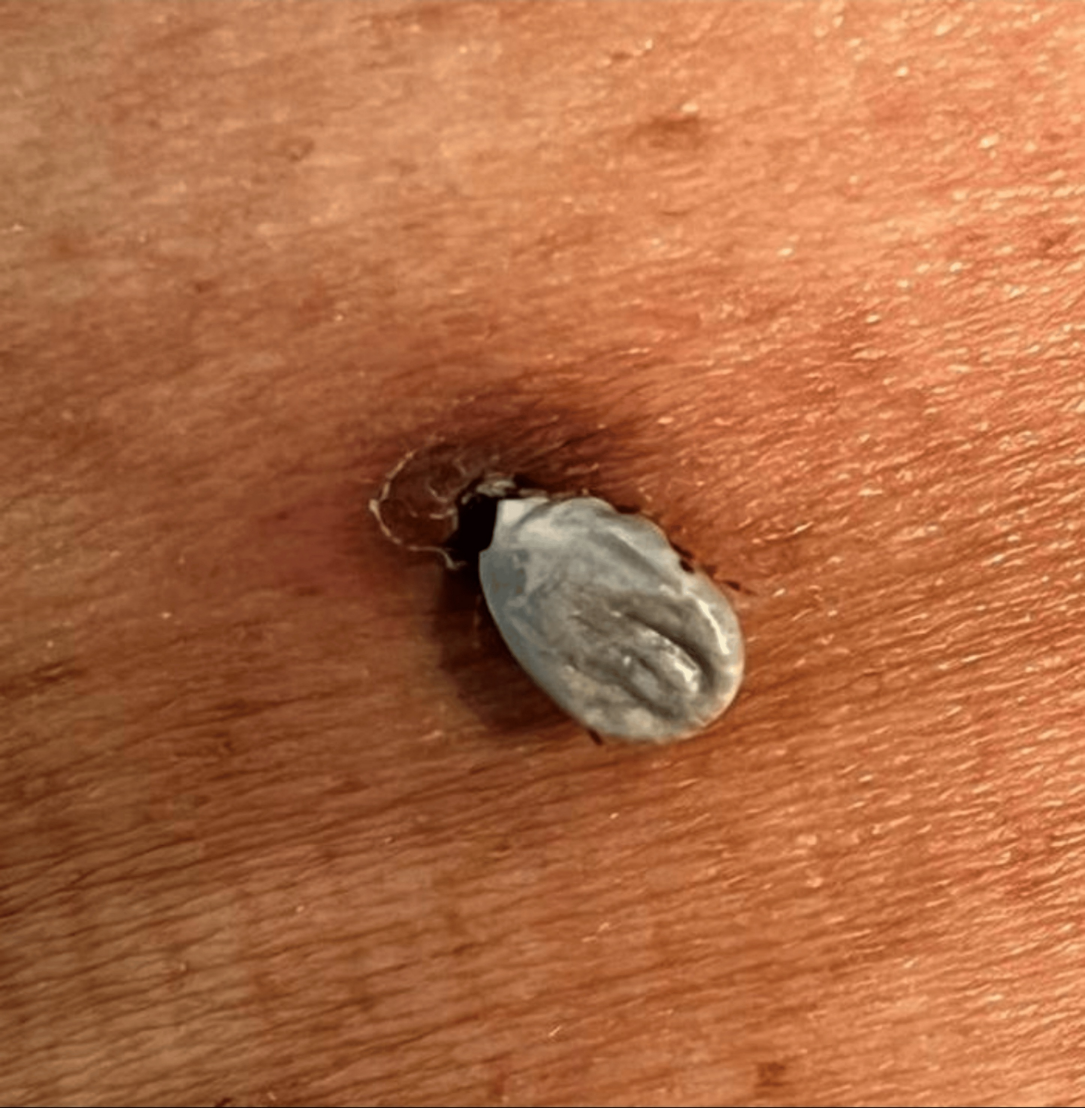
Tick attached to the patient’s left flank. Mild dermatitis is present around the site of attachment, although it is barely visible.

A 70-year-old Saudi male presented to the ED with complaints of localized pain and swelling over his left flank, which had developed over the past two weeks. He reported sustaining a tick bite more than a month earlier following a camping trip in the north of Saudi Arabia. He had not attempted to remove the tick and noted that it had remained in place since that time. He denied experiencing fever, malaise, rash, or any systemic symptoms throughout the duration of the attachment. On physical examination, the patient was afebrile and hemodynamically stable. A live, engorged tick was observed firmly attached to the left flank, surrounded by mild erythema, edema, and localized dermatitis (Figure 1).

**Figure 2:**
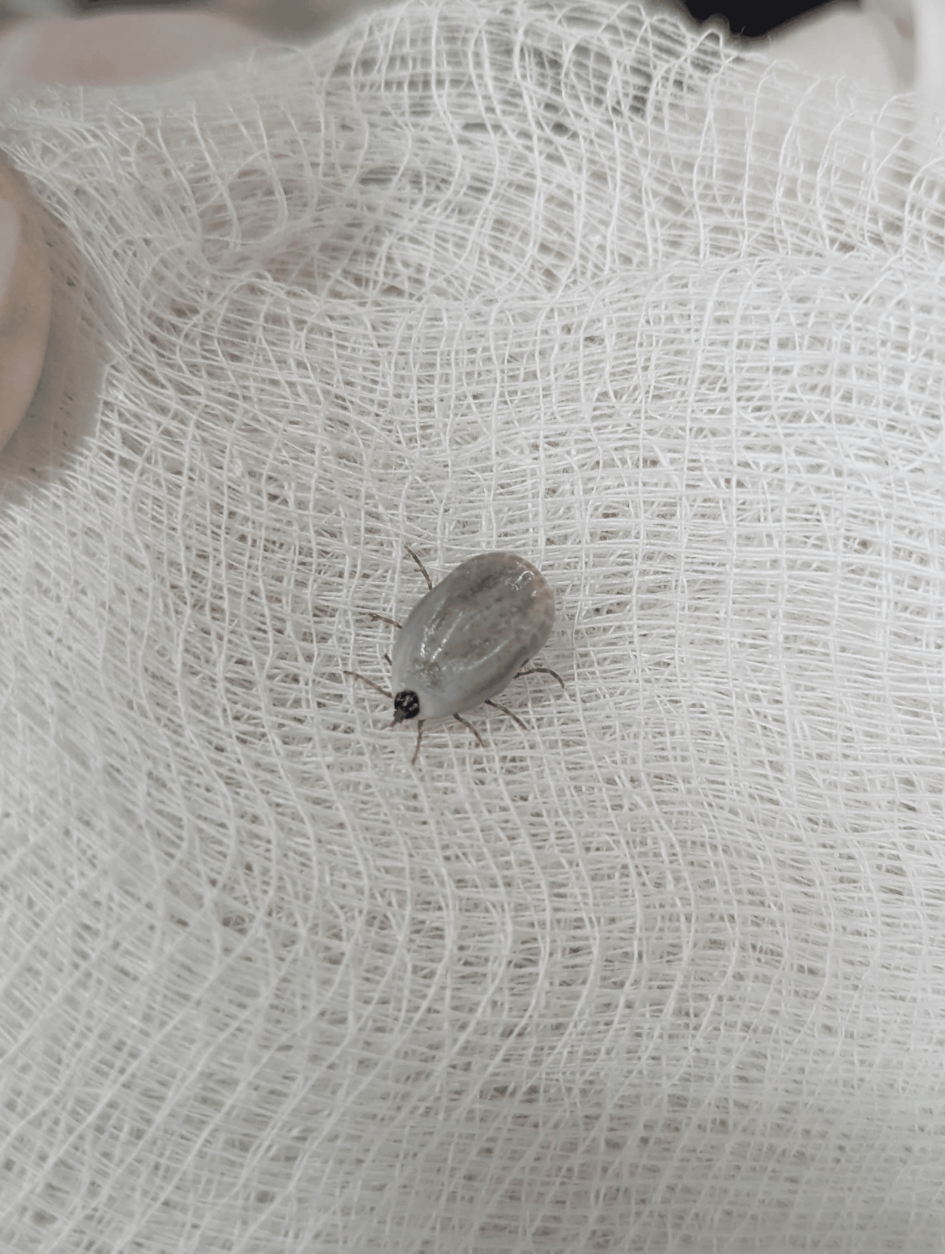
The engorged Rhipicephalus microplus tick following removal.

The lesion measured approximately 0.5 cm in diameter, with no evidence of necrosis, secondary infection, or erythema migrans. The tick was carefully removed intact using sterile tweezers and was subsequently submitted to the regional Public Health Department for species identification (Figure 2).

A punch biopsy of the lesion was also performed to evaluate the local tissue response. Laboratory investigations, including a complete blood count, serum chemistry, and electrolyte panel, were all within normal limits. Table 1 summarizes the patient’s laboratory test results.

**Table 1:** Laboratory investigation results and reference ranges.

Test Description	Test Result	Reference Range
Alanine Aminotransferase (ALT)	19 U/L	0-55 U/L
Aspartate Aminotransferase (AST)	38 U/L	5-34 U/L
Creatine Kinase (CK)	201 U/L	30-200 U/L
Cholesterol	6.0 mmol/L	0-5.18 mmol/L
Creatinine	92 μmol/L	63.6-110.5 μmol/L
Gamma-Glutamyl Transferase (GGT)	89 U/L	12-64 U/L
Iron	25.6 μmol/L	11.6-31.1 μmol/L
Phosphate	1.11 mmol/L	0.8-1.5 mmol/L
Total Protein	88.0 g/L	64-83 g/L
Triglycerides	0.7 mmol/L	0-1.7 mmol/L
Alkaline Phosphatase (ALP)	73 U/L	40-150 U/L
Albumin	53.0 g/L	32-45 g/L
Amylase	84 U/L	20-160 U/L
Calcium	2.46 mmol/L	2.1-2.55 mmol/L
Chloride	101 mmol/L	98-107 mmol/L
Direct Bilirubin	3.9 μmol/L	0-8.6 μmol/L
Magnesium	0.77 mmol/L	0.7-0.91 mmol/L
Potassium	3.90 mmol/L	3.5-4.5 mmol/L
Total Bilirubin	9.5 μmol/L	5.1-20.5 μmol/L
Urea	5.0 mmol/L	3.2-7.4 mmol/L
HbA1c	5.80%	4.6-5.8%
Sodium	139 mmol/L	132-146 mmol/L

**Figure 3:**
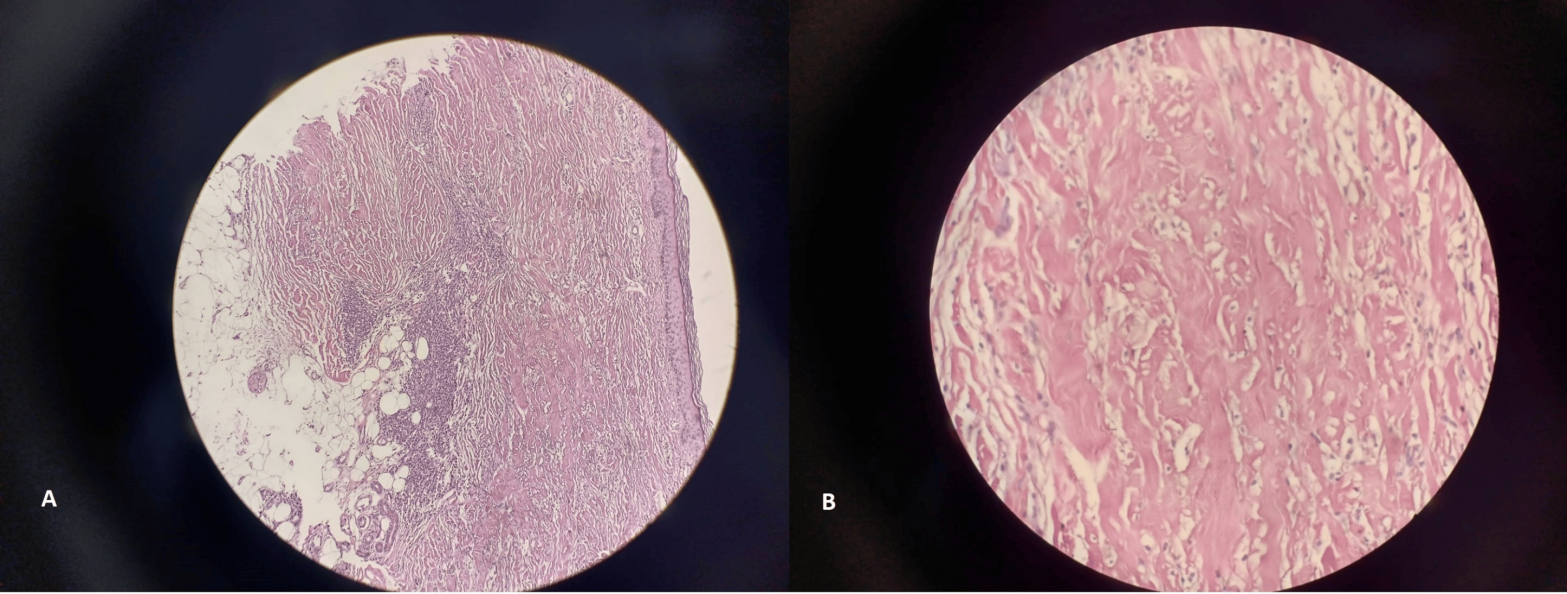
Histopathological findings of the skin biopsy from the tick bite site. (A) Layered alterations observed in the superficial and deep dermis (H&E stain, ×10). (B) Eosinophilic, hyalinized, degenerated collagen bundles consistent with eosinophilic dermatitis (H&E stain, ×40).

Histopathological examination of the skin biopsy revealed nonspecific eosinophilic dermatitis, indicative of a localized hypersensitivity reaction to the tick bite. Figure 3 shows the histopathological findings of the biopsy specimen.

Although the patient remained asymptomatic and the tick was not suspected to be a known vector of human disease, a prophylactic course of doxycycline 100 mg orally once daily for 14 days was prescribed. Identification by the Public Health Department confirmed the tick as *Rhipicephalus microplus* (Asian blue tick). At a two-week follow-up, the patient demonstrated complete healing of the lesion and remained free of systemic complaints or complications.

## Discussion

Tick bites in humans can lead to a spectrum of dermatological and systemic manifestations, influenced by factors such as tick species, duration of attachment, and host immune response. *R. microplus*, commonly known as the cattle tick or Asian blue tick, is predominantly a parasite of livestock, particularly cattle, and is found mainly in tropical and subtropical regions [6]. Human infestations by *R. microplus* are rare, with only a few cases documented in the literature. One such case involved an 82-year-old woman in North India who presented with itchy, red, and watery eyes due to a large number of *R. microplus* larvae attached to her eyelid margins. The infestation was linked to close contact with untreated, heavily infested livestock on her farm. The ticks were manually removed, and treatment with topical antibiotics led to resolution of symptoms [3].

The patient’s presentation, localized eosinophilic dermatitis without fever or other systemic symptoms, aligns with prior observations that non-pathogenic ticks can induce significant inflammatory reactions due to prolonged feeding and host immune responses. Karami M et al. reported a dermatitis outbreak in a five-member family in Babol, Iran, caused by larval hard tick (Ixodidae) infestation. The family experienced significant itching, irritation, and pain, and live larvae were recovered from their skin, clothing, and home environment. This case underscores the clinical significance of larval tick bites even in the absence of systemic illness [5].

*R. microplus* is not a recognized vector for human pathogens, but its prolonged attachment (≥1 month in this case) can trigger localized hypersensitivity reactions, as evidenced by biopsy-confirmed eosinophilic dermatitis. This mirrors findings from other reports where tick saliva, containing immunomodulatory compounds, provoked intense inflammatory responses even in the absence of pathogen transmission [7]. The patient’s age may have contributed to the delayed immune response, as older adults often exhibit reduced cutaneous reactivity to arthropod bites [8]. Notably, the decision to administer prophylactic doxycycline, despite the tick’s non-pathogenic status, reflects clinical caution in high-risk scenarios (e.g., prolonged attachment, elderly patients) [8,9]. However, current guidelines reserve prophylaxis for bites by Ixodes species in Lyme-endemic regions, emphasizing the need for case-by-case risk assessment [8].

This case highlights that even non-pathogenic tick species such as* R. microplus *can remain attached to human hosts for extended periods and provoke localized inflammatory reactions. Despite the absence of systemic illness, comprehensive management, including appropriate tick removal, biopsy of the bite site, and notification of public health authorities, was essential to ensure accurate diagnosis and surveillance. Clinicians should remain vigilant in evaluating tick-bite presentations, regardless of the perceived pathogenicity of the tick species, and ensure adequate follow-up to monitor for delayed complications or evolving symptoms.

## Conclusions

This case demonstrates that even non-pathogenic tick species like *R. microplus* can remain attached to humans for prolonged periods and induce local inflammation. While systemic illness was absent, proper tick removal, biopsy, and public health reporting were essential components of care. Clinicians should maintain a high index of suspicion for atypical tick species in endemic and non-endemic areas alike, provide individualized prophylaxis based on patient risk factors and duration of attachment, and ensure follow-up to monitor for delayed complications. Broader reporting of such uncommon presentations can improve recognition of rare tick-human interactions and inform evidence-based management guidelines.
